# Supplementary material for: Regulation of replication timing in Saccharomyces cerevisiae
Source: PLoS Comput Biol. 2025 Jun 2;21(6):e1013066. doi: 10.1371/journal.pcbi.1013066 (PMC12165382; doi:10.1371/journal.pcbi.1013066)
Supplement: S1 Fig — Beacon Calculus code used for the model. Processes and comments are highlighted in pink and green respectively. The code includes process definitions for firing factors, FF, origins, ORI, and replication forks, FR and FL. Comments within the code are indicated by “\\”. Actions are enclosed within “{}” and are defined as ordered pairs, specifying the action followed by the rate at which it occurs. Handshake communications are denoted by @factor! for sending and @factor? for receiving on the factor channel. Beacon actions are represented by ch! for sending, ch? for checking, and ch? for receiving, all on the ch channel. The values within “[]” following handshake or beacon are transmitted. The code syntax includes “.” for sequential statements, “|” for parallel statements, and “+” for making exclusive choices. Condition gates are represented by “->”. All origin and firing factor processes are initiated from the beginning of the simulation. However, this has been omitted from this representation for conciseness (PDF) [file pcbi.1013066.s001.pdf]

```
fast = 100000; //fast rate
v = 1.4; //fork velocity in kilobases per minute

//process definitions

FF[] = {@factor![0],1}.{dwell,0.05}.FF[];

ORI[i,ch,length,fire] = {@factor?[0],fire}.(FL[i,ch,length]||FR[i,ch,length])
    + {ch?[i],fast};

FR[i,ch,length] = {ch![i],fast}.[i < length] -> {~ch?[i+1],v}.FR[i+1,ch,length];

FL[i,ch,length] = {ch![i],fast}.[i > 0] -> {~ch?[i-1],v}.FL[i-1,ch,length];
```
