# Supplementary figures and images for: Regulation of replication timing in Saccharomyces cerevisiae
Source: PLoS Comput Biol. 2025 Jun 2;21(6):e1013066. doi: 10.1371/journal.pcbi.1013066 (PMC12165382; doi:10.1371/journal.pcbi.1013066)

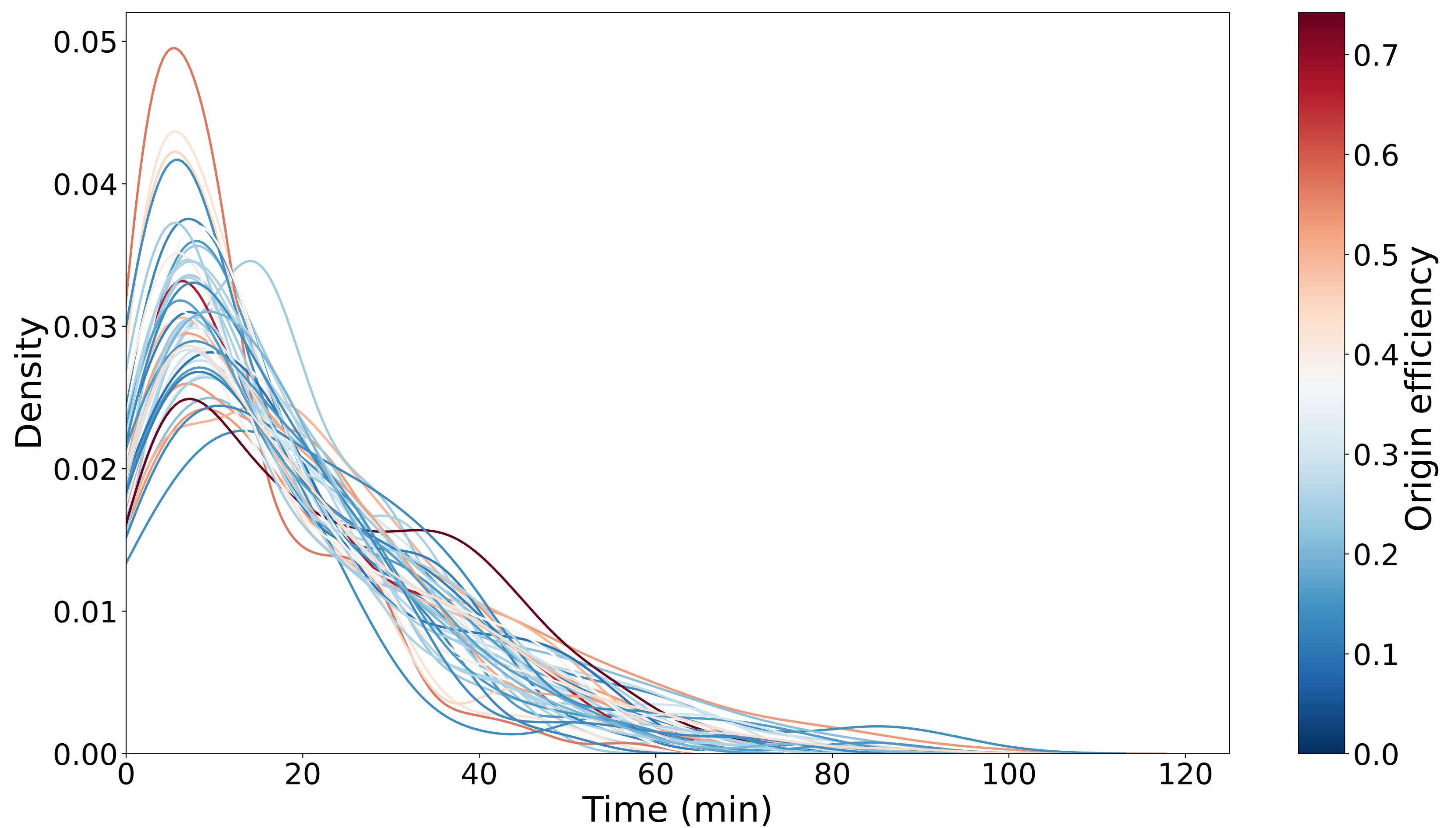

Supplement: S2 Fig — Kernel density plot showing the distributions of origin firing times for all origins on chromosome 2. Each line represents a separate origin, with the colour gradient from blue to red reflecting its efficiency. (PDF) [file pcbi.1013066.s002.pdf]
