# Supplementary material for: Regulation of replication timing in Saccharomyces cerevisiae
Source: PLoS Comput Biol. 2025 Jun 2;21(6):e1013066. doi: 10.1371/journal.pcbi.1013066 (PMC12165382; doi:10.1371/journal.pcbi.1013066)
Supplement: Attachment — Submitted filename: 151737_1_rebuttal_3303666_ss1mqs.pdf [file pcbi.1013066.s003.pdf]

Revision Plan

Review COMMONS

Manuscript number: RC-2024-02743
Corresponding author(s): Michael, Boemo; Francisco Berkemeier

[The "revision plan" should delineate the revisions that authors intend to carry out in response to the points raised by the referees. It also provides the authors with the opportunity to explain their view of the paper and of the referee reports.

The document is important for the editors of affiliate journals when they make a first decision on the transferred manuscript. It will also be useful to readers of the reprint and help them to obtain a balanced view of the paper.

If you wish to submit a full revision, please use our "Full Revision" template. ***It is important to use the appropriate template to clearly inform the editors of your intentions.****]*

1. General Statements [optional]

This section is optional. Insert here any general statements you wish to make about the goal of the study or about the reviews.

We are very grateful to the reviewers for their time and care, and we feel that their comments have greatly improved the manuscript. The attached sheets indicate the revisions we have already made, and those we plan to do, in order to incorporate and address all reviewers' comments to the best of our ability.

2. Description of the planned revisions

Insert here a point-by-point reply that explains what revisions, additional experimentations and analyses are planned to address the points raised by the referees.

Reviewer #1 Major Comments

3. The model currently uses single values for the initiation factor number and recycling rate, though these parameters may vary across cell cycles or under different growth conditions. It is suggested that sensitivity analysis should be added to supplementary materials to explore how the changes of these parameters affect the model output, such as replication time distribution and origin efficiency.

Response:
Sensitivity analysis of how the model fit and validation is affected by using different recycling rates and initial firing factor counts will be conducted.

5. Although the code is open, it is suggested to provide specific instructions or examples of the running code in supplementary materials, so as to facilitate reproduction and application by other researchers.

Response:
The GitHub repository will be updated to enable the running of the entire pipeline. This update will include code for processing replication timing data from Müller et al. (2014) and extracting origin positions from the OriDB. Code will also be provided for writing Beacon Calculus scripts with different parameters and origin firing rates. Instructions on the recommended sequence in which scripts should be executed will also be provided. To enable users to run the model locally on their own computers, a smaller version focused on chromosome 2 will be included in the supplementary information and GitHub repository, along with example input data and expected outputs.

Reviewer #3 Major Comments

3. Currently the code is available as supplemental data. Ideally, the code should be available and provided to run the entire pipeline beginning with the initialization of the origin firing program from the Müller, 2014 data set.

Response:
The GitHub repository will be updated to enable the running of the entire pipeline. This update will include code for processing replication timing data from Müller et al. (2014) and extracting origin positions from the OriDB.

Reviewer #3 Minor Comments

2. The recycling parameter appears to be very important for this model. A sensitivity analysis of the value of 0.05 would be helpful to understand why this value was chosen.

Response:
Sensitivity analysis of how the model fit is affected by using different recycling rates will be conducted.

3. It would be helpful to understand the convergence of the model better. Can the authors provide insight or a plot to better understand why the convergence parameter alpha was chosen as 1.2?

Response:
An explanation of how different values of alpha affect the fitting of the model will be added.

3. Description of the revisions that have already been incorporated in the transferred manuscript

Please insert a point-by-point reply describing the revisions that were already carried out and included in the transferred manuscript. If no revisions have been carried out yet, please leave this section empty.

Reviewer #1 Major Comments

1. This study provides a strong support for the relationship between replication starting point competition and initial factor concentration. However, some predictive conclusions, such as "the origin of high efficiency may not be activated earlier", are still preliminary. Can the author further clarify the scope of these predictions and any potential mechanism in the discussion part to improve the rigor of this study?

Response:
In the discussion, we now emphasize the complexity of predicting origin firing time distributions, which are influenced by multiple interrelated factors beyond efficiency alone.

2. (See section 4)

3. (See section 2)

4. While the authors use mean absolute error (MAE) to assess model fit, it is suggested to add other statistical methods, such as root mean square error or correlation analysis, to further evaluate the model's accuracy and robustness. In addition, this model lacks comparison with other studies on fitting yeast replication time, and it is difficult to evaluate the effect of this model compared with other models from the specific performance.

Response:
We have now included the root mean squared error (RMSE) alongside the mean absolute error (MAE) and R-squared value to compare the simulated replication timing profiles with the experimental data. We agree that we could have been more detailed in comparing our model to other approaches. We have now added a lengthened discussion of this. In some cases, a direct comparison of performance is difficult due to fundamental differences between the approaches, but we have highlighted why this is the case.

5. (See section 2)

6. In Figure 2(a), compared with other chromosomes, the fitting effect of chromosome 1 seems to be not good. Has the author ever thought about the reason? In addition, what is the guiding significance of this model in practical applications, such as online services, forecasting tools, or experiments? Can the author give relevant application examples in this regard?

Response:
Potential explanations for the poorer fit of the replication timing profile for chromosome 1 are now discussed. The y-axis range has also now been set as the same for all subplots in Figure 2a to make the replication timing profiles for each chromosome more easily comparable.
In the discussion, we highlight how the intuitive and flexible nature of the model places it as a valuable tool which could be adapted to predict the effect of different perturbations on DNA replication dynamics.

Reviewer #1 Minor comments

1. Suggestions for Improving Figures:
Figures 2 and 3: It is suggested that the differences between experimental data and model fitting data should be clearly marked by using more distinctive colors or symbols with different shapes in these figures, so as to help readers quickly distinguish between simulation results and experimental observation results. Density Plot in Figure 4: The current color gradient is dense, making it difficult to differentiate activation distributions for different origins. Consider using a broader color gradient or adding a slight separation between each origin's curve to improve readability.

Response:
Keys have been added to Figures 2a and 2b to clarify which lines represent experimental data and which represent the results from our simulations. The colour gradient in Figures 4b and S2 have been changed to make differences in efficiencies between origins more distinguishable. The subset of chromosome 2 origins which are shown in Figure 4b has also been reduced to 1 in 5, rather than 1 in 4, to make it easier to distinguish between different origins.

2. Model Parameter Table:
Adding a table in the Methods section or supplementary materials that summarizes the main model parameters (e.g., number of initiation factors, recycling rate, replication speed) and the basis for each parameter's setting would be helpful. This will allow readers to quickly understand the model setup and provide a reference for future researchers who may wish to use or adjust this model.

Response:
A table summarising the values of model parameters and the rational for each is now provided in the methods section.

3. Citation and Description of Experimental Data:
Clarify the origin and characteristics of the experimental data used, such as the specific details of the replication timing dataset applied for model fitting, and indicate whether the data represents single-cell or population-averaged measurements. This information will help readers better understand the comparison between the model and actual data.

Response:
More details regarding the nature of the experimental replication timing profiles the model was fitted to are now included in the methods section.

4. Background and References:
In the Introduction, consider adding a brief explanation of "Beacon Calculus" to aid non-specialist readers in understanding the novelty and applicability of this method. Adding foundational references for Beacon Calculus would further help readers appreciate the advantages of this approach.
Additionally, in the discussion of the model's suitability for other biological systems, citing some reviews on high-efficiency replication origin analyses would help demonstrate the model's broader applicability.

Response:
A brief explanation of why Beacon Calculus is well suited to modelling DNA replication has been added to the introduction and further details of how Beacon Calculus works are provided in the methods section. We also now highlight that the high conservation of the DNA replication mechanism across eukaryotes means the insights drawn from our model are likely generalizable to other eukaryotes.

Reviewer #2 Major Comments

1. In figure 5, the authors demonstrate that replication dynamics are robust to an increase in the number of available firing factors. However, experimental data from strains in which these limiting factors are overexpressed indicate that replication dynamics are substantially altered (e.g. PMID 22081107 and 23562327) since dNTPs become limiting. So the conclusions of the analysis in figure 5 are at best an oversimplification and at worst rather misleading. If adding dNTPs as a factor that becomes limiting only at higher firing factor concentrations is not technically feasible, the authors should be more circumspect in their description and discussion of the results in figure 5.

Response:
We now discuss the interpretation of the effect of increasing the number of firing factors, given that factors such as dNTP availability are not included in the model.

2. The analysis of replication dynamics appears to exclude origins within the rDNA, which in the average strain account for ~20-25% of all replication origins in S. cerevisiae depending on the origin list chosen. Ignoring this large number of origins likely has a substantial impact on the model: if rDNA origins are intentionally ignored due to the difficulty of modeling repetitive regions or of having multiple identical origins in the competition model, this should be explicitly addressed in the text.

Response:
We now emphasize that our model restricts initiation to specific sites, and we discuss the possibility that initiation sites may be more flexible. Additionally, we note that some low-efficiency origins, such as those in rDNA, have not been included.

Reviewer #2 Minor Comments:

1. Sekedat et al (2010, PMID PMID: 20212525) demonstrated convincingly that replication-fork movement is uniform throughout the genome but are not cited in favor of more recent work.

Response:
The Sekedat et al. (2010) paper has now been cited to support our model's assumption of a constant rate of replication fork movement.

Reviewer #3 Major comments:

1. Can the authors provide some insight into the model's dependency on the Müller, 2014 replication data set? They initialize and converge to this dataset so this paper's findings are highly contingent on treating this data set as ground truth.

Response:
In the discussion, we now highlight that, despite the model's reliance on the Müller, 2014 replication data set for fitting, its ability to reproduce other features of DNA replication demonstrates its ability to reflect DNA replication dynamics more broadly.

2. The authors describe their model as one that simplifies the origin firing mechanisms compared to more complex models. Is there a direct comparison available that can quantify this advantage? Likewise, how does their model compare to a naive discriminative model, such as one that performs peak finding on the replication timing data. For example, the replication fork directionality can be estimated, naively, using a peak finding algorithm. This type of analysis will provide a stronger argument for the usage of their model.

Response:
Quantitative comparisons between our model and other published models are challenging due to differences in underlying assumptions and metrics used to assess goodness of fit. However, we have now added a discussion addressing these challenges and highlighting how our model's design contrasts with that of other models.

3. (See section 2)

4. The authors mention that origin firing factors and their recycling time to be the basis of how this model is constructed. While also describing the recycle time as a general timing delay that

is dependent on a number of reasons such as diffusion and replisome complex formation. Can the authors discuss the limitations of their model towards this simplification?

Response:
Limitations of our model's assumptions of constant recycling rates of firing factors are now discussed, as well as our assumption that the firing rates of origins and the maximum number of available firing factors remain constant between simulations.

Reviewer #3 Minor Comments:
1. The author describes the prediction of 200 active replication forks 22 minutes into S phase. Please discuss why this peak number of active replication forks may have been reached. Is this related to the model configured for the number of firing factors F = 200?

Response:
The timing of the peak in replication fork activity is now explained in the context of the origin firing dynamics and firing factor availability when describing the results for Figure 5a.

2. (See section 2)

3. (See section 2)

4. The authors comment that simulated origin efficiencies were estimated close to zero (6.2%{plus minus}22%). Can the authors comment on the large variability in this estimation (the {plus minus}22%)?

Response:
We now justify the high level of variability in the difference between simulated and experimentally determined origin efficiencies by highlighting that it is similar to the variability in differences when comparing origin efficiencies derived from different experimental techniques.

4. Description of analyses that authors prefer not to carry out

Please include a point-by-point response explaining why some of the requested data or additional analyses might not be necessary or cannot be provided within the scope of a revision. This can be due to time or resource limitations or in case of disagreement about the necessity of such additional data given the scope of the study. Please leave empty if not applicable.

Reviewer #1 Major comment
2. The resolution and accuracy of the model prediction are obvious to all, but the specific generalization ability is still unknown, which makes the further promotion slightly insufficient. Does the author consider conducting additional experiments? To detect the replication time and efficiency in yeast cells with changed levels of key initiation factors (such as Cdc45 or Dpb11).

The empirical data can be compared with the model prediction by editing CRISPR gene or manipulating the initial factor abundance through overexpression vector.

Response:
As this is a theoretical study focused on mathematical modelling, conducting further wet lab experiments would be beyond the scope of this work.
